# Tetraspanin CD151 is a novel prognostic marker in poor outcome endometrial cancer

**DOI:** 10.1038/bjc.2011.80

**Published:** 2011-04-19

**Authors:** M A Voss, N Gordon, S Maloney, R Ganesan, L Ludeman, K McCarthy, R Gornall, G Schaller, W Wei, F Berditchevski, S Sundar

**Affiliations:** 1 Department of Obstetrics and Gynaecology, Cheltenham General Hospital, Gloucestershire, UK; 2 Pan Birmingham gynaecological cancer centre, City Hospital, School of Cancer Sciences, University of Birmingham, Birmingham, UK; 3 Institute of Biomedical research, University of Birmingham, Birmingham, UK; 4 Department of Pathology, Birmingham Womens Hospital NHS trust, Birmingham, UK; 5 Department of Pathology, Cheltenham General Hospital, Gloucestershire, UK; 6 Breast Care Institute (BCI), Munich, Germany

**Keywords:** endometrial cancer, tetraspanin CD151, prognostic marker, type II

## Abstract

**Background:**

Type II cancers account for 10% of endometrial cancers but 50% of recurrence. Response rates to chemotherapy at recurrence are poor and better prognostic markers are needed to guide therapy. CD151 is a small transmembrane protein that regulates cell migration and facilitates cancer metastasis. High CD151 expression confers poor prognosis in breast, pancreatic and colorectal cancer. The prognostic significance of tetraspanin CD151 expression in poor outcome endometrial cancers was evaluated, along with oestrogen receptor (ER), progesterone receptor (PR), p53, human epidermal growth factor receptor -2 (HER-2), and CD 151 staining compared with *α*6*β*1, *α*3*β*1 integrins, and E-cadherin.

**Methods:**

Tissue microarray constructed from 156 poor outcome endometrial cancers, tested with immunohistochemistry and staining correlated with clinicopathological data were used. A total of 131 data sets were complete for analysis.

**Results:**

Expression of CD151 was significantly higher in uterine papillary serous and clear cell carcinoma than in grade 3 endometrioid carcinoma, sarcoma or carcinosarcoma (*P*<0.001). In univariate analysis, age, stage, histology type and CD151 were significant for both recurrence free (RFS) and disease specific survival (DSS). In multivariate analyses, CD151 was significant for RFS and DSS (*P*=0.036 and 0.033, respectively) in triple negative (ER, PR and HER-2 negative) tumours (88/131). The HER-2, p53, ER and PR were not prognostic for survival. There was strong concordance of CD151 with E-cadherin (98%), but not with *α*6*β*1 (35%), *α*3*β*1 staining (60%).

**Conclusion:**

The CD151 is a novel marker in type 2 cancers that can guide therapeutic decisions. CD151 may have an important role in tumourigenesis in some histology types.

Endometrial carcinoma is the most common neoplasm of the female genital tract in the United States and Europe with a worldwide estimated incidence in 2008 of 287 000 cases and 74 000 deaths from the disease (Ferlay *et al*, 2010). Endometrial cancer has increased by over 40% in the United Kingdom since 1993, to an incidence of 7536 in 2007 and 1741 deaths in 2008. (Cancer Research UK, 2010) This increase is attributed to increased life expectancy, tamoxifen use and obesity (Bray *et al*, 2005).

Endometrial cancer can be divided into oestrogen-dependent adenocarcinoma with endometrioid morphology (type I) and non-oestrogen-dependent adenocarcinoma (type II) predominantly with serous papillary or clear cell but also grade III endometrioid morphology (Bokhman, 1983; Rose, 1996). Molecular alterations involved in the development of endometrioid endometrial carcinomas are different from those of non-endometrioid carcinomas (Lax and Kurman, 1997). Type I carcinomas show more microsatellite instability, and mutations in the PTEN, K-RAS, PIK3CA and *β*-catenin genes. Alterations of p53, STK15, p16, E-cadherin and HER-2 are more frequently found in type II cancers (Kohler *et al*, 1996; Risinger *et al*, 1998; Maxwell *et al*, 2001; Morrison *et al*, 2006).

Type I cancers are diagnosed at an early stage and have a good prognosis with >95% 5-year survival. Although type II carcinomas only account for 10–20% of all endometrial malignancies (Bansal *et al*, 2009), they are responsible for ∼50% of all relapses (Carcangiu and Chambers, 1992, 1995; Levenback *et al*, 1992) and a low 5-year, all stage, overall survival rate of 35% (Singh *et al*, 2008). Chemotherapy regimes in advanced stage and recurrent endometrial cancer have been disappointing, with a meta-analysis demonstrating a translated improvement in progression free survival by 1 month (pooled OR) with a concomitant significant impact on quality of life from intensive chemotherapy regimes (Humber *et al*, 2005). Few markers exist that can provide useful clinical guidance for decision making, particularly, as is usually the case, where the tumour marks negative for oestrogen (ER) and progesterone (PR) receptors.

CD151 is one of 33 proteins in the mammalian tetraspanin superfamily, which has emerged as a key player in malignancy, the immune system, during fertilisation and infectious disease processes (Hemler, 2008). The CD151 is a widely expressed transmembrane protein, which is abundantly present on the surface of various types of normal epithelial cells that make contact with the basement membrane. Tetraspanins have a key role in the formation of tetraspanin-enriched microdomains (TERM or TEM), which also contain a number of other transmembrane receptors (e.g., integrins, receptors tyrosine kinases). Laminin-binding integrins (i.e., *α*3*β*1, *α*6*β*1, *α*6*β*4, *α*7*β*1) are most commonly associated with TEMs and it has been shown that tetraspanins regulate integrin-dependent adhesion strengthening, cell spreading and migration (Yanez-Mo *et al*, 1998; Sincock *et al*, 1999; Lammerding *et al*, 2003). Integrin-tetraspanin complexes are critical for regulating cell invasiveness and controlling intercellular interactions, both of which have a critical role during the metastatic progression (Stipp, 2010). In breast cancer, CD151 expression is increased in patients with invasive ductal carcinomas and this correlates with higher tumour grade and node metastasis. Importantly, CD151 was found to be a significant marker for poor outcome (Novitskaya *et al*, 2010; Sadej *et al*, 2010). The CD151 has also been validated as a significant prognostic marker of outcome in lung and prostate cancers (Tokuhara *et al*, 2001; Ang *et al*, 2004).

In this study, we investigate a cohort of endometrial cancers, characterised clinically by poor histological features and anticipated poor survival. The prognostic significance of CD151 expression was assessed and compared with clinicopathological features and previously used markers ER, PR, p53 and HER-2 in patients with poor outcome endometrial cancer. We also compared expression of CD151 with its binding integrin partners –*α*6*β*1, and *α*3*β*1, and with E-cadherin in the tissue microarray.

## Patients and methods

### Patients and specimen characteristics

Upon approval from the Southmead research ethics committee (REC ref. 07/H0102/64), archived paraffin-embedded tissue blocks were collected from all women who underwent treatment for International Federation of Gynaecology and Obstetrics (FIGO) stage IA–IV uterine cancers at the Gloucestershire NHS Foundation Trust Hospitals between 1997 and 2002. Histology types grade 3 endometrial adenocarcinoma (G3 EEC), uterine papillary serous cancer (UPSC), clear cell carcinoma (CC), carcinosarcoma (MMMT) and sarcoma were selected. Clinicopathological information and survival data were abstracted from a regional prospectively collected clinical database held at the South West Public Health Observatory (SWPHO; Bristol, UK). A total of 85.9% of patients underwent total abdominal hysterectomy with bilateral salpingo-oophorectomy and peritoneal washings, in some women pelvic node sampling was performed if they had been recruited to the ASTEC trial (Kitchener *et al*, 2009). This was consistent with surgical practice in the United Kingdom, in the study time period. Patients without gross peritoneal disease and lymphadenectomy were staged according to the extent of uterine involvement. Adjuvant platinum based chemotherapy or pelvic radiation was given at the discretion of the multidisciplinary team according to local guidelines. No patient received chemotherapy or radiotherapy before surgery. Patients were kept under surveillance for 5 years, longer follow-up details were abstracted from hospital records from other attendances. Clinical endpoints were survival or death of disease or other causes or date of recurrence as recorded in the hospital notes. Data were cross-referenced against cancer registry data to ensure accuracy.

### Tissue microarray construction

Hematoxylin- and eosin-stained slides of tumours were reviewed to confirm original diagnosis and two separate representative areas of tumour marked on a corresponding paraffin block. Tissue biopsies of 0.6 mm diameter were taken from each donor block and mounted in a recipient block using a tissue array machine. (Beecher Instruments, Sun Prairie, WI, USA) Cores were set at 1 mm intervals, two cores per case, to decrease the risk of aberrant results due to tumour heterogeneity. Completed array blocks were sealed for 10 min in a 60°C oven. The tissue array was monitored by hematoxylin- and eosin-stain and in case of lack of visible tumour a duplicate biopsy processed.

### Immunohistochemistry

The 4 *μ*m paraffin sections from the array blocks were used. Mouse anti-CD151 monoclonal antibody (mAb) The NCL-CD151 (Novocastra, Newcastle, Upon Tyne, UK) was used at dilution 1 : 50 (Berditchevski *et al*, 1997). Positive and negative controls were samples of breast cancer and normal breast, respectively. After 16 h antigen retrieval with 1 × 1 mM EDTA pH 8.0, 0.1% Tween 20 buffer, immunostaining was carried out using standard avidin-biotin-peroxidase complex method in the Shandon Sequenza Immunostaining Station. (Thermo Electron Corp. US, Waltham, MA, USA).

Cell surface adhesion molecules goat anti-human *α*3 integrin subunit (Santa Cruz, Santa Cruz, CA, USA, sc-6588, antibody concentration 0.67 *μ*g ml^−1^; dilution 1 : 300), rabbit anti-human *α*6 integrin subunit (Santa Cruz, sc-10730, antibody concentration 0.5 *μ*g ml^−1^; dilution 1 : 400) and mouse anti-E-cadherin (clone NCH-38, Dako (Copenhagen, Denmark), antibody concentration 0.1 *μ*g ml^−1^; dilution 1 : 2000) were used. The W-CAP TEC buffer pH 8.0 (Bio-Optica Milano s.p.a, Milan, Italy) was used in three-step preparation. Sections were quenched in 0.3% H_2_O_2_ in distilled water before primary antibody incubation at 4°C for 16 h. The R.T.U Vectastain Universal Quick kit (Vector laboratory, Burlingame, CA, USA), and Dako Real Envision DAB chromagen and solution (product: K5007, Dako) were used to detect both *α*3*β*1 and *α*6*β*1. Sections of ductal carcinoma *in situ* (with skin) were used as a positive tissue control.

Antibodies to oestrogen receptor (ER) (1D5 clone, Dako, Code M7047, total protein concentration 11.9 g l^−1^, dilution of 1 : 35), progesterone receptor (PR) (PgR 636 clone, Dako, Code M3569, dilution of 1 : 35) and p53 (D-07 clone, Dako, Code M7001, total protein concentration 11.9 g l^−1^, dilution 1 : 50) were used. Heat induced epitope retrieval was carried out using Antigen Access Unit (A. Menarini Diagnostics, Workingham, UK). Dako Auto Stainer universal platforms were used for staining.

The expression of HER-2 was evaluated in an accredited laboratory using Pathway HER-2 (Clone CB11) on the BenchMark XT automated system (Ventana Medical Systems Inc., Tucson, AZ, USA) and HER-2 antibody: A0485 clone (polyclonal antibody, Dako, HercepTest, dilution of 1 : 400) was used with known positive and negative breast cancer cell cultures included as controls.

Positive staining for CD151 was defined by crisp cytoplasmic and partly membranous staining, ER and PR by clear nuclear staining, p53 by unequivocal strong nuclear staining, HER-2 by clear membranous staining; *α*3*β*1, *α*6*β*1 integrins by cytoplasmic and membranous staining and E-cadherin by strong membranous staining.

### Evaluation of immunohistochemical staining

All markers were evaluated independently, using light microscopy at × 400 magnification by two pathologists blinded to the data on at least two separate occasions. In event of intra-observer or inter-observer variation a consensus score was decided after examination at a multi-headed microscope. CD151, ER, PR and p53 were scored in a semi-quantitative manner incorporating both intensity of staining and distribution. Staining intensity (I) was graded as 0 (no staining), 1 (weak), 2 (moderate), or 3 (strong). The proportion (P) of cells (0–100%) with the observed staining intensity was recorded. A score (histologic or H-score) was determined as the product of the intensity and proportion using the formula (H=I × P) (Budwit-Novotny *et al*, 1986; Katz *et al*, 1990; Wilder *et al*, 2004). H-score=(1 × percentage of cells stained at intensity category 1)+(2 × percentage of cells stained at intensity category 2)+(3 × percentage of cells stained at intensity category 3). H-score between 0 and 300 was obtained where 300 was equal to 100% of tumour cells stained strongly. H-score ⩾150 was considered positive for the above markers. HER-2 scores were evaluated by a pathologist (GS) as per established guidelines (Wolff *et al*, 2007). Cases were scored from 0–3 and considered positive when >1.

### Statistical analysis

Fisher's exact test was used for association analyses of tumour types or stages with proportion of positive expression. Survival data were analysed with Kaplan–Meier estimator and Cox proportional hazards model. A *z*-test was used to test statistical significance of each coefficient in the model. Deaths due to causes other than endometrial cancer were excluded from analyses of disease specific survival (DSS) or recurrence free survival (RFS). All statistical analyses were carried out using R (http://www.r-project.org/). Two-sided *P*-values less than 0.05 were considered statistically significant. The ‘REMARK’ criteria of the National Cancer Institute were used in design, analysis and interpretation (McShane *et al*, 2005).

## Results

A total of 156 patients that fitted the inclusion criteria were treated in 1997–2002. Clinical and pathological features are summarised in Table 1. Seventy-eight patients died during the observation period, 60 related to endometrial cancer and 18 related to other causes. A total of 64 adverse events were recorded, which included recurrent disease as well as deaths related to endometrial cancer. Three histological groups were formed for analysis: G3 EEC=group 1; USPC+CC=group 2 and sarcoma+MMMT+mixed tumours=group 3. Of 156 patients included in this study, 131 patients (83.97%) had archived paraffin embedded tissue and complete follow-up data available for analysis.

Mean follow-up time was 4.01 years (range, 0.01–11.79 years). Mean all-stage-, all-histology- overall survival (OS) was 4.01 years (range 0.01–11.79 years), DSS (114 of 131) was 4.267 years (range 0.01–11.79) and RFS (114 of 131) was 4.108 years (range 0.01–11.79). Patients with low stage (I–II) had a DSS of 5.318 years whereas patients with advanced stage (III–IV) lived significantly shorter with a DSS of 2.24 years (hazard ratio (HR) 4.373, CI 2.55–7.49, *P*<0.001) (Table 3).

**Figure 1:**
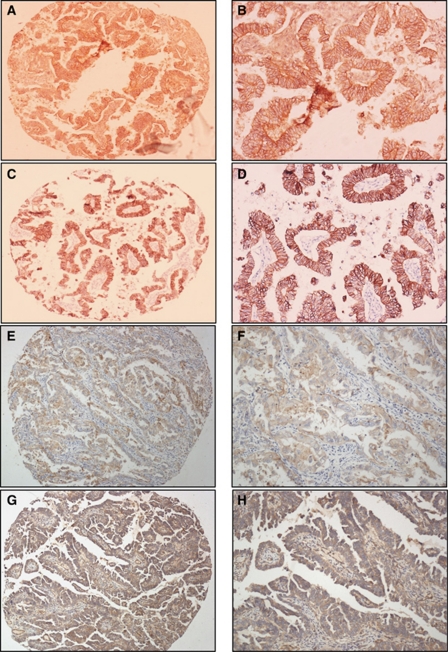
Immunohistochemistry with antibodies to CD151, HER-2, integrins *α*6*β*1, *α*3*β*1 and E-cadherin. **A** and **B** – CD151 ( × 100 and × 200, respectively); **C** and **D** – E-cadherin ( × 100 and × 200, respectively); **E** and **F** – integrin *α*3*β*1 ( × 100 and × 200, respectively); **G** and **H** – integrin *α*6*β*1 ( × 100 and × 200, respectively).

Figure 1 shows immunohistochemistry with antibodies to CD151, HER-2, E-cadherin, and *α*3*β*1, *α*6*β*1 integrin subunits. CD151 scored positive in 98.5% of USPC+CC cases, but less than 50% of G3 EEC or sarcoma+MMMT+mixed group. CD151 expression was significantly raised in USPC+CC tumour types compared with G3 EEC (*P*<0.001) and sarcoma+MMMT+mixed (*P*<0.001). Expression of CD151 was highest in ‘triple-negative’ tumours as compared with the rest of the cohort (*P*<0.001). A detailed analysis of CD151, HER-2, ER, PR and p53 expression by stage and histology is presented in Table 2. CD151 showed no specific association with tumour stages. HER-2 positivity (>1) was seen in only eight cases (6.11%).

**Figure 2:**
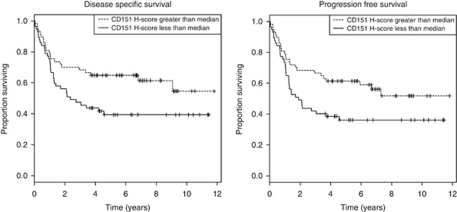
Kaplan–Meier survival curves demonstrating effect of CD151 expression on DSS and RFS in the entire cohort. For DSS, the hazard ratios of CD151 greater than median is 1.816 (95% confidence interval 1.060–3.109, *P*=0.02746) and for RFS is 1.773 (95% confidence interval 1.062–2.961, *P*=0.02647) indicating that there is a significant survival benefit in the group overexpressing CD151.

In univariate analyses age, stage, expression of CD151 and histology type, specifically sarcoma/MMMT/mixed group, were significant factors impacting on DSS and RFS (Table 3). Low CD151 expression was associated with significantly worse DSS (HR 1.816, *P*=0.02) and RFS (HR 1.773, *P*=0.02) when compared with strong expression, this is represented in Figure 2. HER-2, ER, PR and p53 expression were not significantly associated with survival. The association of the diminished expression of CD151 with reduced DSS (HR 2.94, *P*<0.001) and RFS (HR 2.54, *P*<0.001) was even stronger in the triple negative (ER, PR and HER-2-negative) subgroup (*n*=88). In multivariate analyses age, stage and tumour type maintained significance. CD151 maintained prognostic significance in multivariate analysis for the triple negative subgroup (DSS, *P*=0.033, RFS, *P*=0.036) but not for the entire cohort (Table 4). Oestrogen receptor, PR, p53 and HER-2 were not significant factors influencing survival.

The *α*3*β*1, *α*6*β*1 and E-cadherin were also investigated in one tissue microarray and compared with CD151 expression. Strong staining with *α*6*β*1 integrin was seen in four cores, in 14 cores for *α*3*β*1 strong staining and in 26 cores for E-cadherin staining. There was strong concordance (98%) of CD151 and E-cadherin staining, with only 1 of 43 tumours showing discordance in staining (i.e., cores positive for CD151 were positive for E-cadherin staining and cores negative for CD 151 were negative for E-cadherin staining). Only 15 of 43 cores showed concordance with *α*6*β*1and CD151 staining (35%) and 26 of 43 cores (60%) showed concordance between *α*3*β*1 and CD151 staining (Figure 1).

## Discussion

This is the first study to evaluate the staining and prognostic significance of tetraspanin CD151 in endometrial carcinoma. We selected tetraspanin CD151 as a marker based on studies in breast cancer, which demonstrated its prognostic utility in a subgroup of invasive ductal carcinomas (Sadej *et al*, 2009; Novitskaya *et al*, 2010). Our results show that CD151 is an independent marker for DSS and RFS in poor outcome endometrial carcinoma by univariate analysis and for a triple negative subgroup of patients by multivariate analysis. CD151 is also differentially expressed, with highest expression in the UPSC and CC histology types. Contrary to our expectation, we found that high CD151 expression is positively correlated with improved survival. Thus, this is the first report, which suggests that expression of this tetraspanin protein in tumours may prevent transition to a more malignant phenotype.

The strengths of this study are the long duration of follow-up and high numbers of poor prognostic types of tumours. Our cohort includes G3 endometrioid endometrial cancers, which are usually not included into type II endometrial cancers. However, they fit the criteria in so far as they demonstrate more aggressive behaviour and are of significantly poorer prognosis (58% 5 year survival) than low-grade endometrioid cancers (Bokhman, 1983). In addition, analyses of survival outcome between histological groups I and II show no difference in RFS or DSS (data not shown). Additionally we included sarcomas, carcinosarcomas and uterine tumours of mixed histology for the same reason – they are also poor prognosis tumours with currently no adjuvant treatment that has shown to clearly improve survival (Sutton *et al*, 2000; Deniaud-Alexandre *et al*, 2001; Weitmann *et al*, 2002; Giuntoli *et al*, 2003; Livi *et al*, 2003; Toyoshima *et al*, 2004). Although the tissue set contains divergent histology types, clinically these tumours result in poor outcome and justify being investigated together.

Increased expression of CD151 also correlates with advanced tumour grades and poor prognosis in other tumours. Ang *et al*, (2004) showed that overall survival was reduced in prostate cancer tumours where CD151 was overexpressed . This was consistent with findings in lung cancer (Tokuhara *et al*, 2001). In colon cancer, changes in expression of CD151 appear to be more complex. An early report has described that increased expression levels of this tetraspanin correlated with a more advanced stage of the disease (Zijlstra *et al*, 2008). However, a more recent study has found that expression of CD151 protein was reduced in human colon cancers compared with surrounding normal tissue, in which it is strongly expressed on the basal and lateral surfaces of epithelial cells (Chien *et al*, 2008). The authors hypothesised that intra-tumoural activation of hypoxia-inducible factor 1 led to inhibition of CD151 expression and this repressed the function of E-cadherin (Chien *et al*, 2008), thereby dramatically reducing cell-to-cell adhesion and thus increasing invasion and metastasis. Interestingly, it was also found that the expression level of CD151 in the metastatic lesions was increased when compared with primary colon cancer tissues.

Although the exact biochemical function of CD151 is still unknown, evidence shows that it is involved in signal transduction (Yauch *et al*, 1998; Zhang *et al*, 2001), cell adhesion (Hasegawa *et al*, 1998), and motility (Testa *et al*, 1999). In relation to tumour metastasis, experiments using anti-CD151 mAb have established that this tetraspanin may contribute to an early step in the formation of secondary metastatic lesions by mediating invasiveness of primary tumour cells into surrounding stromal tissues and vascular intravasation (Testa *et al*, 1999). More recently, we have shown that CD151 can also regulate recruitment of breast cancer cells to the lungs and growth of the metastatic lesion (Sadej *et al*, 2010). Thus, tetraspanin CD151 may be involved in various aspects of the metastatic cascade.

Although our results strongly suggest that CD151 may have an important role in tumourigenesis in some histology types of endometrial cancer, the mode of its action remains unknown. Holcomb *et al*, (2002) examined E-cadherin in endometrial carcinoma and found that papillary serous and clear cell carcinomas show significantly reduced E-cadherin expression in comparison with endometrioid tumours (*P*=0.01). Thus, one possibility might be that CD151 counteracts the metastatic progression of endometrial cancer by stabilizing E-cadherin based cell-cell interactions. In this regard, it is noteworthy that we found a strong correlation in the expression levels of E-cadherin and CD151 in cancer samples. Alternatively, CD151 may act through laminin-binding integrins, its main molecular partners in tetraspanin microdomains, by strengthening interactions of endometrial cells with laminin components of the basement membrane. Detailed immunohistochemical analyses of endometrial cancer tissues will be necessary to address this issue in the future.

So far, treatment options in type II cancers are largely limited to surgical- and/or chemotherapy regimen, which have not altered the poor prognosis of these tumours.

There is an unmet clinical need for robust prognostic markers that can help in guiding therapeutic decisions in these tumour types, particularly at recurrence and at advanced stage. This study suggests that, in patients with CD151 positive tumours, survival is significantly better than those with CD151 negative tumours. This could be highly clinically relevant as even in a triple negative marker group, CD151 is highly prognostic of both DSS and RFS. A larger prospective study of CD151 in this group of cancers is now being established to confirm these findings. An enhanced understanding of the molecular basis of these tumours will also aid in the development of novel, targeted therapies.

## Figures and Tables

**Table 1:** Clinicopathological data

**Clinical data**	**Patients (*n*=156)**
Age, mean (s.d., range)	68.2 (11.4, 37–89)
	
*FIGO stage,* n *(%)*	
I	87 (55.77)
II	14 (8.97)
III	35 (22.4)
IV	20 (12.8)
	
*Histologic type,* n *(%)*	
G3 EEC	78 (50.0)
UPSC	33 (21.15)
Clear cell	9 (5.8)
MMMT	18 (11.5)
Sarcoma	14 (8.97)
Mixed histology	4 (2.6)
	
*Myometrial invasion,* n *(%)*	
⩽50%	58 (37.2)
>50%	94 (60.3)
Missing	4
	
*Hysterectomy*	
Yes	134 (85.9)
No	22 (14.1)
	
*Lymphnodes*	
Taken	32 (20.51)
Not taken	124 (79.49)
	
*Chemotherapy,* n *(%)*	
Yes	28 (17.9)
No	128 (82.1)
	
*Radiotherapy,* n *(%)*	
Yes	64 (41.0)
No	92 (59.0)
	
*Outcome*	
Alive without disease	73 (46.8)
Alive with disease	4 (2.6)
Died of disease	61 (39.1)
Death of other causes	18 (11.5)
Observation time mean (range)	148 months (0.1–11.07 years)
Complete data for analysis	131 (83.97%)
Lost for follow-up or missing tissue-samples	25 (16.03%)

Abbreviations: G3 EEC=grade 3 endometrioid endometrial carcinoma; FIGO=International Federation of Gynaecology and Obstetrics; MMMT=malignant mixed mullerian tumour; UPSC=uterine papillary serous cancer.

**Table 2:** Clinicopathological features of CD151, ER PR, p53 and HER-2 expression by immunohistochemistry

	**Patients *n***	**CD151 positive *n* (%)**	**ER positive *n* (%)**	**PR positive *n* (%)**	**P53 positive *n* (%)**	**HER-2 positive *n* (%)**
*Stage*						
I	71	45 (63.3)	12 (16.90)	6 (8.45)	15 (21.12)	3 (4.22)
II	14	5 (35.7)	2 (14.3)	0	3 (21.4)	2 (14.3)
III	28	12 (42.8)	3 (10.7)	2 (7.1)	9 (32.1)	1 (3.6)
IV	18	10 (55.5)	4 (22.2)	2 (11.1)	4 (22.2)	2 (11.1)
(I–II)	85	50 (58.8)	14 (16.5)	6 (7.1)	18(21.2)	5 (5.9)
(III–IV)	46	22 (47.8)	7 (15.2)	4 (8.7)	13 (28.3)	3 (6.5)
						
*Histology*						
Group I	68	25(36.76)	15 (22.06)	3 (4.4)	13 (19.1)	3 (4.4)
USPC	31	30 (96.7)	6 (19.4)	4 (12.9)	11 (35.4)	4 (12.9)
CC	7	7 (100)	0	1 (14.3)	1 (14.3)	1 (14.3)
Sarcoma	13	4 (30.8)	0	0	5 (38.5)	0
MMMT	9	1 (11.1)	0	2 (22.2)	0	0
Tumours mixed histo	3	0	0	0	1 (33.3)	0
Group II	38	37 (97.4)	6 (15.8)	5 (13.2)	12 (31.6)	5 (13.2)
Group III	25	5 (20)	0	2 (8)	6 (24)	0

Abbreviations: CC=clear cell carcinoma; ER=oestrogen receptor; MMMT=malignant mixed mullerian tumour; PR=progesterone receptor; UPSC=uterine papillary serous sarcinoma.

ER, PR, p53 and CD151 are regarded as positive when H-score ⩾150. The HER-2 is regarded positive when score >1. Group I comprised of G3 EEC, group II comprised of UPSC and CC, group III comprised of MMMT, sarcoma and mixed mesodermal tumours.

**Table 3:** Univariate analysis of DSS and RFS

	**DSS**			**RFS**		
	**HR**	**95% CI**	***P***	**HR**	**95% CI**	***P***
Age	1.055	1.02–1.08	<0.001	1.056	1.03–1.08	<0.001
Stage (I, II *vs* III, IV)	4.373	2.55–7.49	<0.001	3.965	2.37–6.62	<0.001
						
*Histology* a						
Group II *vs* group I	1.069	0.55–2.067	0.84	1.056	0.56–1.96	0.86
Group III *vs* group I	2.926	1.58–5.40	<0.001	2.577	1.41–4.69	0.001
CD151	1.816	1.06–3.10	**0.02**	1.773	1.06–2.96	**0.02**
CD151 (in ER, PR and HER-2 negative tumours)	2.94	1.50–5.74	**<0.001**	2.541	1.37–4.71	**0.008**
HER-2	1.541	0.55–4.26	0.402	1.408	0.51–3.88	0.506
P53	1.283	0.71–2.32	0.409	1.112	0.62–1.99	0.72
ER	0.815	0.38–1.72	0.591	0.851	0.419–1.72	0.655
PR	0.962	0.34–2.66	0.94	0.864	0.31–2.38	0.77

Abbreviations: CI=confidence interval; DSS=disease specific survival; ER=oestrogen receptor; HR=hazard ratio; PR=progesterone receptor; RFS=recurrence free survival.

Univariate analysis of age was performed in a linear manner; therefore, there is no referent variable.

a Univariate analysis of histology carried out with endometrioid histology as reference. Significant *P*-values for CD151 are in bold.

**Table 4:** Multivariate analysis of DSS and RFS

	**DSS**			**RFS**		
	**HR**	**OR – 95%CI**	***P*-value**	**HR**	**OR – 95%CI**	***P*-value**
Age	1.0600	1.0335–1.087	<0.001	1.0595	1.0343–1.085	<0.001
Stage	5.0119	2.8090–8.942	<0.001	4.5366	2.6132–7.876	<0.001
						
*Histology*						
Group II	1.0603	0.4215–2.667	n.s.	1.0757	0.4534–2.552	n.s.
Group III	2.7967	1.3878–5.636	0.004	2.4431	1.2438–4.799	0.0095
CD151	1.4304	0.6424–3.185	n.s.	1.5535	0.7332–3.292	n.s.
CD151 (in ER, PR, HER-2 negative tumours)	3.168	1.1005–9.123	**0.033**	2.792	1.0682–7.299	**0.036**

Abbreviations: CI=confidence interval; DSS=disease specific survival; ER=oestrogen receptor; HR=hazard ratio; n.s.=non-significant; OR=odds ratio; PR=progesterone receptor; RFS=recurrence free survival.

A multivariate regression model was designed utilizing a backwards elimination. Significant *P*-values for CD151 are in bold.
